# Unexpected compound reformation in the dense selenium-hydrogen system

**DOI:** 10.1038/s43246-025-00899-9

**Published:** 2025-08-21

**Authors:** Huixin Hu, Mikhail A. Kuzovnikov, Hannah A. Shuttleworth, Tomas Marqueño, Jinwei Yan, Israel Osmond, Federico A. Gorelli, Eugene Gregoryanz, Philip Dalladay-Simpson, Graeme J. Ackland, Miriam Peña-Alvarez, Ross T. Howie

**Affiliations:** 1 Center for High Pressure Science and Technology Advanced Research, Shanghai, China; 2 School of Physics and Astronomy, Centre for Science at Extreme Conditions, University of Edinburgh, Edinburgh, UK; 3 SHARPS (Shanghai Advanced Research in Physical Sciences), Shanghai, China; 4 CNR-INO, Instituto Nazionale di Ottica, Sesto Fiorentino, Italy; 5 Key Laboratory of Materials Physics, Institute of Solid State Physics, Hefei, China

**Keywords:** Structure of solids and liquids, Phase transitions and critical phenomena, Chemical physics

## Abstract

The H_2_Se molecule and the van der Waals compound (H_2_Se)_2_H_2_ are both unstable upon room temperature compression, dissociating into their constituent elements above 22 GPa. Through a series of high pressure-high temperature diamond anvil cell experiments, we report the unexpected formation of a novel compound, SeH_2_(H_2_)_2_ at pressures above 94 GPa. X-ray diffraction reveals the metallic sublattice to adopt a tetragonal (*I*4_1_/*amd*) structure with density functional theory calculations finding a small distortion due to the orientation of H_2_ molecules. The structure comprises of a network of zig-zag H-Se chains with quasi-molecular H_2_ molecular units hosted in the prismatic Se interstices. Electrical resistance measurements demonstrate that SeH_2_(H_2_)_2_ is non-metallic up to pressures of 148 GPa. Investigations into the Te-H system up to pressures of 165 GPa and 2000 K yielded no compound formation. The combined results suggest that the high pressure phase behavior of each chalcogen hydride is unique and more complex than previously thought.

## Abstract (web summary)

High-pressure studies of chalcogen hydrides reveal complex phase behaviors, challenging existing assumptions about their stability and composition. Here, the authors discover a novel compound, SeH_2_(H_2_)_2_, at pressures above 94 GPa, characterized by a unique tetragonal structure, highlighting the intricate nature of high-pressure chemistry and its implications for material science.

## Introduction

Since the discovery of high-temperature superconductivity in the sulfur-hydrogen system, immense experimental and theoretical efforts have been made to understand its behavior at extreme densities, revealing surprising complexity^1–9^. It is understood that under compression, the simplest sulfur hydride, H_2_S, can react further with hydrogen to form the molecular compound (H_2_S)_2_H_2_, which undergoes a series of phase transitions, before the molecules dissociate becoming covalent bonded H_3_S^10,11^. Above 150 GPa, H_3_S adopts a body-centered cubic structure (space group $Im\bar{3}m$) that exhibits a superconducting transition temperature, *T*_*c*_, of 203 K, whilst below 140 GPa, there is a rhombohedral distortion forming the $R\bar{3}m$ phase with a reduced T_*c*_^1–3^. Similar to the S-H system, the other chalcogen hydrides, Se-H and Te-H are expected to form covalently bonded compounds that exhibit superconductivity; however, neither have been experimentally explored at the predicted synthesis pressures^12–14^.

Hydrogen is known to react with the heavier chalcogens to form H_2_Se and H_2_Te. Whilst H_2_Te is unstable under ambient conditions, H_2_Se is stable and can be readily synthesized from its constituent elements at high temperature^15^. Upon compression, H_2_Se solidifies into face-centered cubic phase I ($Fm\bar{3}m$) at 1.5 GPa before transitioning to a hydrogen-bonded structure analogous to H_2_S-IV at 12 GPa^16^. On further compression, H_2_Se-IV dissociates into elemental Se and molecular H_2_ at 24 GPa at 300 K^16^. It was also observed that when H_2_Se is synthesized in an excess of H_2_, (H_2_Se)_2_H_2_, forms above 4.2 GPa^16,17^. This is analogous to (H_2_S)_2_H_2_, formed in the H-S system, and adopts a tetragonal structure, with space group *I*4/*mcm*^10^. Similar to pure H_2_Se, (H_2_Se)_2_H_2_ is increasingly sensitive to irradiation, decomposing into its constituent elements at 24 GPa when exposed to laser emission or x-rays^16^ and persist to  ~39.5 GPa at 170 K^17^.

Previous theoretical predictions of the H-Se system found *Cccm*-H_3_Se (a slightly distorted variant of *I*4/*mcm*-(H_2_S)_2_H_2_) to be stable to higher pressures, decomposing above 67 GPa^13^. At pressures above 90 GPa, H_3_Se is predicted to reform with space group $Im\bar{3}m$ (isostructural to superconducting $Im\bar{3}m$-H_3_S), and exhibit a T_*c*_ of 131 K at 200 GPa. Another study predicted two energetically stable superconducting phases above 120 GPa: *C*2/*m*-HSe_2_ and $Im\bar{3}m$-H_3_Se, with T_*c*_’s ranging between 5 and 116 K, respectively^12^. Although the maximum predicted critical temperature is comparatively lower than that of H_3_S^1^, the phases are predicted to have lower synthesis pressures^12^. Similarly, stable compositions are predicted in the H-Te system above 100 GPa, however, due to the heavier atomic mass and weaker electronegativity, the theoretical compositions (H_4_Te and H_5_Te_2_) are distinctly different to that of either the H-Se and H-S systems^14^. Interestingly, these compounds contain a combination of quasi-molecular H_2_ units and linear H_3_ units, with H_4_Te becoming superconducting below 104 K at 170 GPa^14^. Despite these predictions, neither the H-Se nor the H-Te system has been experimentally investigated above 40 GPa.

Here we have explored the selenium-hydrogen system in a series of laser-heated diamond anvil cell experiments up to 154 GPa and  ~1500 K. We find that up to pressures of 22 GPa, the only stable compositions are H_2_Se and (H_2_Se)_2_H_2_, both of which decompose into elemental selenium and H_2_ upon further compression. However, heating the decomposition products above 94 GPa promotes the formation of another compound, SeH_2_(H_2_)_2_. X-ray diffraction measurements reveal the selenium sublattice to have *I*4_1_/*amd* symmetry. Density functional theory (DFT) calculations find a stable structure consistent with the experimental data, but with a small distortion due to H_2_ orientations at zero temperature. Raman spectroscopy measurements of this new structure show signatures associated with H_2_ molecular units and Se-H covalent bonds, whilst calculations of the electron localization function (ELF) demonstrates a network of H-Se bonded zig-zag chains. Electrical resistance measurements show SeH_2_(H_2_)_2_ to be non-metallic, consistent with our calculations. Studies of the Te-H system did not yield any stable compounds up to 165 GPa.

## Results and discussion

### Synthesis and structural characterization of selenium polyhydrides

**Fig. 1:**
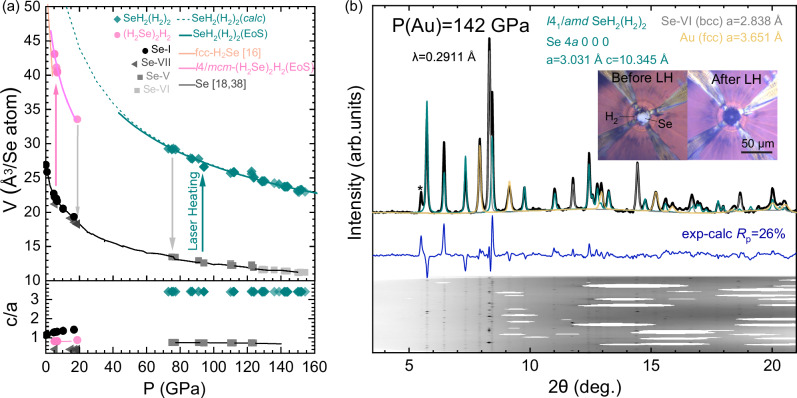
*P*-*V* compression curve and x-ray diffraction pattern of SeH_2_(H_2_)_2_. **a** Volumes per selenium atom as a function of pressure (upper panel) and c/a ratio with pressure (bottom panel). Cyan symbols represent SeH_2_(H_2_)_2_, pink symbols represent (H_2_Se)_2_H_2_, and gray symbols represent Se. The cyan solid line is the Birch-Murnaghan equation of state (EoS) with *V*_0_ = 69(3) Å^3^/Se atom, *B*_0_ = 15(2) GPa, and fixed *B*_0_' = 4. The literature V(P) data for fcc-H_2_Se^16^ and Se^18,38^ are shown by solid orange and black lines, respectively. The pink solid line represents the fitted second-order Birch-Murnaghan EoS for (H_2_Se)_2_H_2_. The dashed cyan line represent the DFT calculated volume of SeH_2_(H_2_)_2_. The pink and cyan arrows indicate the minimal pressure at which a reaction was observed upon compression, whilst the gray arrows indicate the decomposition conditions. **b** Representative x-ray powder diffraction pattern and the results of the Rietveld refinement of *I*4_1_/*amd*-SeH_2_(H_2_)_2_ (cyan) at 142 GPa (*a* = 3.031 Å, *c* = 10.345 Å). The experimental data are shown in black and the refinement residuals are shown in blue. The calculated contributions from unreacted Se-VI and the gold pressure standard are shown in gray and yellow, respectively. An unidentified impurity peak is marked with an asterisk. The raw diffraction (cake) image is shown below the residuals. The Insert: photomicrographs of the sample before and after laser heating.

**Fig. 2:**
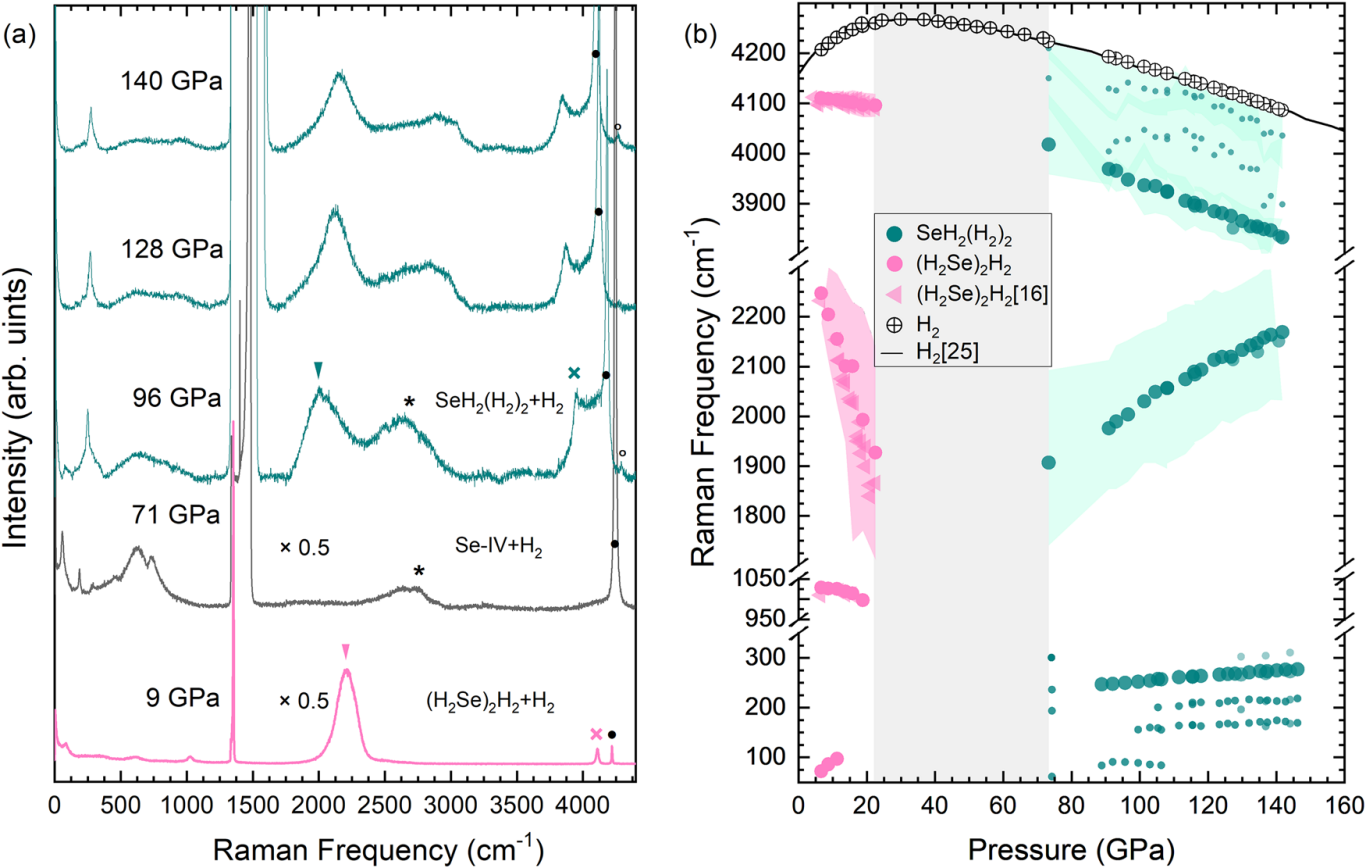
Raman spectra and Raman frequencies as a function of pressure for (H_2_Se)_2_H_2_ and SeH_2_(H_2_)_2_. **a** Representative Raman spectra of (H_2_Se)_2_H_2_ (pink), Se-IV (gray) and SeH_2_(H_2_)_2_ (cyan). Triangles indicate Se-H vibrational modes, crosses indicate H-H modes, asterisks indicate the second order modes from the diamond anvil, solid black dots indicate unreacted excess H_2_. Black open circles represents a minor amount of (CH_4_)_3_(H_2_)_25_ formed during laser heating through the reaction between H_2_ and the diamond anvil^39,40^. Scaling factors are given for spectra at 9 and 71 GPa. **b** Raman shift as function of pressure of (H_2_Se)_2_H_2_ (pink circles from this study and pink triangles from ref. ^16^), SeH_2_(H_2_)_2_ (cyan circles) and unreacted excess H_2_ (black circles). The black solid line is pure H_2_ taken from ref. ^25^. The colored shaded areas represent the full width at half maximum (FWHM) of the Raman bands.

High-purity selenium powder (99.99%) was loaded into the diamond-anvil cells and subsequently gas loaded with research grade hydrogen (99.9995%) at 0.2 GPa (a complete description of the experimental and computational methods is given in the “Methods” section below). Following a previously reported synthesis route^16,17^, Se was laser-heated in a hydrogen environment at 0.4 GPa until it reacted to form H_2_Se. Upon further compression above 4.2 GPa, (H_2_Se)_2_H_2_ formed with space group *I*4/*mcm* (*a* = 7.326 Å and *c* = 6.116 Å, at 6 GPa), with the volume per Se atom in good agreement with the previously determined equation of state (EoS) (see Fig. 1a)^16^. Typical x-ray diffraction patterns of Se-I and (H_2_Se)_2_H_2_ are shown in Supplementary Fig. 1. The formation of (H_2_Se)_2_H_2_ was also confirmed by Raman spectroscopy measurements through the observation of both the H_2_Se stretching band and an intramolecular vibrational band corresponding to quasi-molecular H_2_ within the structure (Fig. 2a). The frequency of the H-H mode is downshifted compared to pure H_2_ by ~111 cm^−1^ at 9 GPa. The H_2_Se stretching band rapidly reduces in frequency upon compression (see Fig. 2b) and at 16 GPa, (H_2_Se)_2_H_2_ begins to decompose into H_2_ and Se. At pressures above 22 GPa, x-ray diffraction and Raman measurements show only a mixture of solid H_2_ and Se (Fig. 1a, Fig. 2a and Supplementary Fig. 1). Laser heating was performed in 4–5 GPa intervals up to pressures of 94 GPa, with no further reaction between Se and H_2_ observed, whilst compression up to 150 GPa at 300 K also did not yield a reaction. The selenium precursor underwent the expected transformations from Se-IV to Se-V at  ~82 GPa and to Se-VI at 127 GPa^18^.

Laser heating samples between 94 GPa and 154 GPa (the highest pressure reached in this study) results in a dramatic change in the x-ray diffraction pattern and the Raman spectrum (Fig. 1b and Supplementary Fig. 2). Rietveld refinement of the crystal structure revealed a *β*-Sn-type metal lattice (*I*4_1_/*amd*) with *a* = 3.031 Å, *c* = 10.345 Å, and *V* = 23.76 Å^3^ per Se atom, at 142 GPa, although we note that this *c*/*a* ratio is approximately six times larger than in *β*-Sn (3.3 vs 0.54). The *V*(*P*) dependence of the new hydride can be fitted well with a second-order Birch-Murnaghan EoS with parameters listed in Supplementary Table 1 (dark cyan curve in Fig. 1a)^19^, indicating that this phase has a pressure-independent composition. The volume of this new compound is marginally smaller than the sum of the volumes of Se and 3H_2_, suggesting that it has H/Se = 6^18,20^. Upon decompression, we find the compound to be stable down to 74 GPa, below which it decomposes into Se-V and H_2_ (Fig. 1a).

**Fig. 3:**
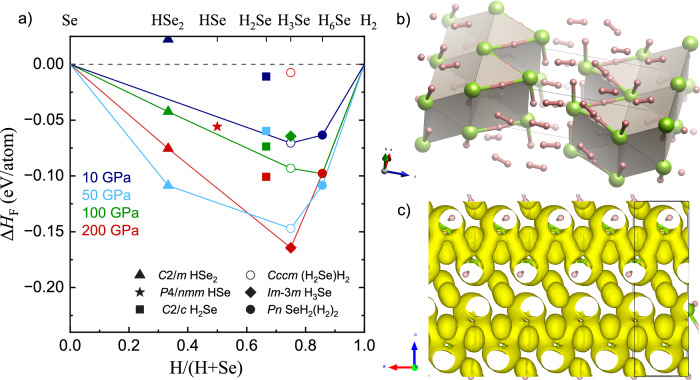
Convex hull diagram of the H-Se system and structural models of SeH_2_(H_2_)_2_. **a** Formation enthalpies (Δ*H*_F_) predicted by DFT for various Se-H compounds relative to constituent elements (Se and H_2_) at 10 GPa (dark blue), 50 GPa (light blue), 100 GPa (green) and 200 GPa (red). The convex hulls are shown by solid lines. **b** Crystal structure model of *Pn*-SeH_2_(H_2_)_2_ at 100 GPa. Green and pink spheres represent Se and H atoms, respectively. (**c**) *Pn*-SeH_2_(H_2_)_2_ crystal structure at 100 GPa with Se and H atoms represented as green and and pink spheres, respectively. ELF isosurfaces (ELF = 0.6) shown in yellow are associated with Se-H-Se interactions and H_2_ molecules. Se-H chain-like bonding is clearly observed along the a-axis, in addition to Se-H bonds coming out of the page in the b-axis. Further isosurfaces are shown in Supplementary Figs. 7 and 8.

In order to determine the hydrogen positions, we investigated the H_6_Se compound through DFT calculations as implemented in the CASTEP code^21^. Despite extensive state-of-the-art structure searching, previous theoretical works have never considered this composition^12,13^. Rather than structure searching, we adopted a molecular dynamics approach, starting with four highly unstable H_6_Se octahedral “molecules" arranged in *I*4_1_/*amd* symmetry, and running at 300 K with aggressive thermostating. After a few picoseconds the structure stabilized, forming eight rotating H_2_ molecules, four Se ions and eight extra protons located between the seleniums, the molecular content is consistent with other high pressure hydrides^22^. On relaxation, we find a stable structure with pseudo-tetragonal symmetry, *Pn* (Fig. 3b and Supplementary Table 2), with *a* = 3.04 Å, *b* = 3.03 Å, *c* = 10.17 Å and *β* = 90.08° at 142 GPa, which is in agreement with x-ray diffraction measurements. A larger molecular dynamics simulation with a double-sized unit cell found no further distortions.

### Stability and bonding of SeH_2_(H_2_)_2_

To further understand the phase stability, we calculated the convex hull of enthalpy of different H to Se ratios at 10, 50, 100 and 200 GPa (Fig. 3a). Previous DFT work has shown some sensitivity to exchange correlation treatment^13^, and we find that with the rSCAN functional^23^, the H_6_Se compound is theoretically stable at pressures between 10 and 200 GPa. The topological analysis of the ELF^24^ can offer further insight into the chemical bonding in the structure. At 100 GPa, electrons in the H_2_ molecules, located in prismatic Se interstitial sites, show extremely high localization (ELF > 0.97), in agreement with a single covalent bond. The remaining atoms form zigzag chains (- SeH - H - SeH - H -) with hydrogen atoms lying outwith the chain in SeH bonds (1.46 Å at 120 GPa) and others approximately midway between Se atoms with separations along the chain (1.53–1.63 Å at 120 GPa) (see Supplementary Table 3). High ELF values (above 0.9) are found at the hydrogen positions in the short SeH bond in the b-direction. Along the chain, in the a-c plane, the -Se-H-Se can be seen as a tube of electrons with ELF above 0.6 (see Fig. 3c), much lower than is usually regarded as a covalent bond, but well above the free-electron value of 0.5. These results indicate that there is a Se-H bond delocalization along the a–c plane, providing the structure with a unique character of Se-H chains. As such, we hereafter refer to the H_6_Se compound as SeH_2_(H_2_)_2_.

This bonding is experimentally evidenced by intense Raman bands corresponding to Se-H and H-H vibrational modes. Interestingly, the frequency of the Se-H stretching band of the SeH_2_(H_2_)_2_ compound at  ~100 GPa is comparable to that observed for Se-H vibrational modes in (H_2_Se)_2_H_2_ at 16 GPa (2030 cm^−1^ and 2026 cm^−1^, respectively). However, the pressure-dependency of the frequency is the opposite (see Fig. 2b). While the Se-H stretching mode in (H_2_Se)_2_H_2_ softens with pressure (indicative of the instability of the H_2_Se molecule), in SeH_2_(H_2_)_2_ the Se-H stretching mode hardens upon compression. The experimental Raman results are consistent with our calculations, whereby a distinct group of six Se-H stretch modes overlapping in the range 1800–2300 cm^−1^ are found, stiffening and mixing with other H-motions as pressure increases (Supplementary Fig. 3).

The Raman bands at 3832–4018 cm^−1^ are indicative that the Se-H bonded network of SeH_2_(H_2_)_2_ hosts H_2_ units (see Fig. 2b). Our calculations predict three double degenerate modes corresponding to H-H stretches, spread across 200 cm^−1^, with the lowest frequency mode having the highest Raman intensity. This is in good agreement with experiments whereby we see an intense low frequency mode together with lower intensity, unresolvable bands at frequencies closer to that of pure H_2_ (see Fig. 2a and Supplementary Fig. 3). We fitted the experimental Raman spectra assuming three separate Raman active modes in this range, and plot their frequencies in Fig. 2b with large and small filled cyan circles for the strongest and two weaker modes, respectively. We also show the full width at half maximum as a shaded area to cover the full frequency regime of the bands. Interestingly, the frequency of the most intense H-H mode lies almost on a linear extrapolation for that observed for (H_2_Se)_2_H_2_ below 20 GPa. Upon compression, the vibrational mode softens at a rate of −2.65 cm^−1^ per GPa, similar to that of phase-I of pure H_2_ (−2 cm^−1^ per GPa) in the same pressure range. At 146 GPa, the mode reaches a frequency of 3832 cm^−1^, which is comparative to pure H_2_ in phase-III at a pressure of 217 GPa^25^. The calculated H-H bond lengths are around 0.74 Å, reaching a minimum value at 120 GPa (see Supplementary Table 3).

The pressure-induced recombination after decomposition into constituent elements is a rarity in hydride systems^26–28^. Silane (SiH_4_) has been shown to amorphize above 60 GPa, coinciding with the dissociation of the tetrahedral molecules^28^. Above 90 GPa, there is recrystallization into two polymeric structures, one of which (*I*4_1_/*a*) has a Si atom bonded to 8 hydrogen atoms. The behavior observed in the Se-H system system bears similarities to the Si-H system, whereby molecular H_2_Se dissociates upon compression, recombining at higher pressure to form a compound with polymeric-like H-Se chains. The Se-H stretching mode behaves markedly similar to the Si-H mode, where the frequency of the Si-H vibron decreases prior to decomposition, and hardens upon recombination^28^. Furthermore, the frequency of the Si-H vibron before dissociation is close to the frequency upon recombination.

### Electrical measurements of SeH_2_(H_2_)_2_

It was previously predicted that the H-Se system would yield stable phases which would be metallic and exhibit superconductivity, namely H_3_Se which is isostructural to superconducting H_3_S^1,3,12,13^. Motivated by this, to explore the electronic properties of SeH_2_(H_2_)_2_ we have performed electrical resistance measurements (see “Methods” section for further details). Before laser heating, we observed the resistance of unreacted Se drop from  ~2 Ω in phase I at 12 GPa to 7 mΩ in phase V at 120 GPa (see Supplementary Fig. 4). After laser heating, the resistance markedly increased on the order of MΩs suggesting that SeH_2_(H_2_)_2_ is non-metallic. To rule out that the high resistance was due to electrical contact issues, we performed repeated experiments, all of which exhibited an increase in resistance after heating cycles. Calculations of the electronic density of states at pressures of 70 GPa and 120 GPa, indeed find SeH_2_(H_2_)_2_ to possess a band gap (see Supplementary Fig. 5). At 120 GPa, the band gap is calculated to be 0.5 eV, typical of a semiconductor, consistent with our observations that the sample transforms from highly reflective metallic selenium, to SeH_2_(H_2_)_2_, which is black in appearance (Fig. 1b). At 70 GPa, the molecular H_2_ bonds form distinct bands 8–13 eV below the Fermi energy, separated from the delocalized electrons in the Se-Se and Se-H chains. As pressure increases, the delocalised bands broaden and merge with the H_2_ states, so at 120 GPa there is no gap in the eDoS between the H_2_ bond and the valence band. There is significant occupation of the H_2_ bonding states (see Supplementary Tables 3 and 4) whereas the absence of any distinctive Se-Se covalent bonds suggests that the valence region is better described as “delocalized" rather than “polymeric". Given that Raman spectroscopy demonstrates that SeH_2_(H_2_)_2_ contains H_2_ molecules, it is unsurprising that the compound is non-metallic, as the vast majority of known hydrides with molecular H_2_ units are insulators.

We have performed multiple heating cycles up to pressures of 158 GPa and do not observe the formation of the aforementioned predicted H_3_Se phases^12,13^. It could be that higher pressures are required to synthesize such phases. Indeed our calculations find $Im\bar{3}m$-H_3_Se is a stable phase above 200 GPa and may coexist with SeH_2_(H_2_)_2_. This would indicate that the synthesis pressure of H_3_Se is considerably higher than H_3_S and also yields a lower superconducting T_*c*_.

### Studies of the tellurium-hydrogen system

Given the unexpected synthesis of SeH_2_(H_2_)_2_, we have also explored whether the tellurium-hydrogen system could produce a similar compound. Although H_2_Te is unstable at ambient conditions, it is predicted that stable phases could form at pressures above 100 GPa^14^. Interestingly, one of these compounds, H_4_Te is predicted to be stable at 162 GPa and like SeH_2_(H_2_)_2_, contains quasi-molecular H_2_ units. We have explored the synthesis of such compounds through multiple laser heatings of Te embedded in a H_2_ medium at pressures between 4 and 165 GPa. Our x-ray diffraction and Raman spectroscopy measurements indicate that no Te-H compound is stable within this pressure regime.

## Conclusions

We have explored compound formation in the dense selenium-hydrogen system up to 154 GPa. While (H_2_Se)_2_H_2_ forms at 4 GPa, it is markedly unstable upon compression, decomposing into its constituent elements above 22 GPa. We observe no reaction between selenium and H_2_ up to 94 GPa. However, heating at this pressure induces the unexpected synthesis of SeH_2_(H_2_)_2_. The structure is comprised of a network of H-Se zig-zag chains and quasi-molecular H_2_ units, unique to this chalcogen hydride. Intriguingly, no phases containing quasi-molecular hydrogen have been found in the H-S system above pressures of 100 GPa, while superconducting phases, analogous to bcc-H_3_S, did not form in the H-Se system up to 154 GPa. Furthermore, we found no compound formation in the tellurium-hydrogen system up to 165 GPa. It will be of great interest to observe if similarities will emerge at higher pressures (e.g., above 200 GPa) or if Te-H compounds will form; however, these conditions still pose a challenge to experiments.

## Methods

### Sample preparation

We used symmetric-type diamond anvil cells with wide apertures combined with ultra-low fluorescence diamonds of the Boehler-Almax design. The diamonds had culet diameters ranging from 40 to 70 μm bevelled at 8° to a diameter of 300 μm. We used either rhenium or composite insulating gaskets made of a MgO mixture with low-viscosity epoxy for resistance measurements.

High-purity selenium powder (99.99%, Sigma Aldrich) was loaded into the diamond-anvil cells and subsequently gas loaded with research grade hydrogen (99.9995%, BOC) at 0.2 GPa. Hydrogen was always in excess, serving both as a reagent and the pressure-transmitting medium. Pressure was determined either through the EoS of gold in x-ray diffraction measurements^29^, or by Raman measurements of the stressed diamond and cross-referenced with the intramolecular vibrational (vibron) frequency of excess H_2_^25,30^.

### X-ray diffraction measurements

Angular-dispersive powder x-ray diffraction experiments were performed at the Extreme Conditions Beamline (ECB, P02.2) at PETRA-III, Hamburg, Germany^31^, at the ID15-b beamline at the ESRF, Grenoble, France^32^, and at the 13-IDD beamline at Advances Photon Source, Argonne, USA^33^. Typically, the cell was oscillated around *ω* axis by  ±15°, and the 2D diffraction image was collected for 20–30 s. At P02.2, we used an incident X-ray beam with *E* = 42.4 keV (*λ* ≃ 0.2911 Å),focused to a  ~2 × 2 μm spot using Kirkpatrick-Baez mirrors. Diffraction images were recorded using Perkin Elmer XRD1621 detector with a sample-to-detector distance (SDD) of 424 mm or 404 mm, as calibrated with a CeO_2_ standard. At ID15-b an incident beam with *E* ≃ 30.2 keV (*λ* ≃ 0.4097 Å) was focused to a  ~1 μm spot. The diffraction patterns were collected with EIGER2 X 9M detector with SDD ≃ 181 mm, calibrated using a Si powder standard. At 13IDD an incident beam with *E* ≃ 42.0 keV (*λ* ≃ 0.2952 Å) was focused onto a  ~2 × 2 μm, and a Pilatus3X 1M detector was used for data acquisition. The SDD ≃ 207 mm was calibrated with a LaB_6_ standard. The SDD, detector orientation and wavelength calibration, primary processing, azimuthal integration and background subtraction were done with the DIOPTAS v0.5.5 software^34^. Phase analysis and Rietveld refinements were done with the POWDERCELL 2.4 program^35^ and FullProf.2k (Version 7.00)^36^.

### Raman spectroscopy measurements

Raman spectroscopy measurements were conducted using 514.5 or 532.0 nm excitation wavelengths via a custom-built micro-focused Raman systems in 180° backscattering geometry. To study (H_2_Se)_2_H_2_, the laser power was kept below 10 mW to prevent sample decomposition. Samples were laser heated *in house* by directly coupling to a yttrium-aluminum-garnet continuous wave laser with wavelength *λ* = 1064 nm.

### Electrical resistance measurements

For resistance measurements, the electrodes were sputtered onto the diamond surface with a Korvus HEX magnetron sputtering system through a custom-made mask in van der Pauw geometry. A layer of about 200 nm of tungsten was deposited first to ensure good adhesion, and a 100 nm gold layer was deposited on top of it to decrease the resistance of the electrodes and to protect tungsten from reacting with the hydrogen. The distance between the opposing electrodes was about 5–10 μm. Before sample loading, the resistance of the electrodes was checked to be infinity, and otherwise, the diamond culets were etched chemically to remove metals deposited between the electrodes. Typically, the resistance between any of the electrodes was of the order of 500 Ω measured with a two-probe technique with the pressurized selenium sample before laser heating.

### Computational methods

In order to determine the hydrogen positions, we investigated the compound through DFT calculations using CASTEP (C19 ultrasoft pseudopotentials, rSCAN exchange correlation, 463 eV plane wave cutoff and 27 k-points on a Monkhorst-pack grid)^21^. We ran ab initio molecular dynamics at 300 K starting with H_6_Se “octahedral molecules” in the experimentally determined *I*4_1_/*amd* structure. The heavy Se remain close to their symmetry positions, the hydrogens rearranging to create two H_2_ molecules per formula unit which rotate at 300 K. We then performed geometry optimization on a series of snapshots from the molecular dynamics, which produced a most stable structure with Se_4_H_24_ composition and *Pc* symmetry (Space group 7, also *Pn*). At low pressures there are distinctive H_2_Se molecules with Se-H bond lengths between 1.4 and 1.5 Å, which becomes increasingly asymmetric with pressure until by 100 GPa the structure is closer to SeH + 2(H_2_) with the remaining hydrogen midway between two Se atoms. The symmetry-breaking comes from the hydrogens, so the simulated powder pattern is indistinguishable from the experimental data. We also calculated phonon frequencies using the method of finite displacements^37^ and find the *Pn* structure to be dynamically stable, with no imaginary phonon frequencies (Supplementary Fig. 6).

## Supplementary information

Supplemental Material

## Data Availability

The data that support the findings of this study are available from the corresponding author upon reasonable request.
