# Supplementary material for: Unexpected compound reformation in the dense selenium-hydrogen system
Source: Commun Mater. 2025 Aug 21;6(1):193. doi: 10.1038/s43246-025-00899-9 (PMC12370533; doi:10.1038/s43246-025-00899-9)
Supplement: Supplementary file 1 — Supplemental Material [file 43246_2025_899_MOESM1_ESM.pdf]

Supplemental Material for "Unexpected compound reformation in the dense selenium-hydrogen system"

Huixin Hu[1], Mikhail A. Kuzovnikov[2], Hannah A. Shuttleworth[2], Tomas Marqueño[2], Jinwei Yan[2], Israel Osmond[2], Federico A. Gorelli[1], Eugene Gregoryanz[2,3,4], Philip Dalladay-Simpson[1], Graeme J. Ackland[2], Miriam Peña-Alvarez[2], Ross T. Howie[1,2*]

[1]Center for High Pressure Science and Technology Advanced Research, 1690 Cailun Road, Shanghai, 201203, China.
[2]School of Physics and Astronomy, Centre for Science at Extreme Conditions, University of Edinburgh, Edinburgh, EH8 8AQ, United Kingdom.
[3]SHARPS (Shanghai Advanced Research in Physical Sciences), 68 Huatuo Road, Shanghai, 201203, China.
[4]Key Laboratory of Materials Physics, Institute of Solid State Physics, Hefei, 230031, China.

*Corresponding author(s). E-mail(s): ross.howie@ed.ac.uk;

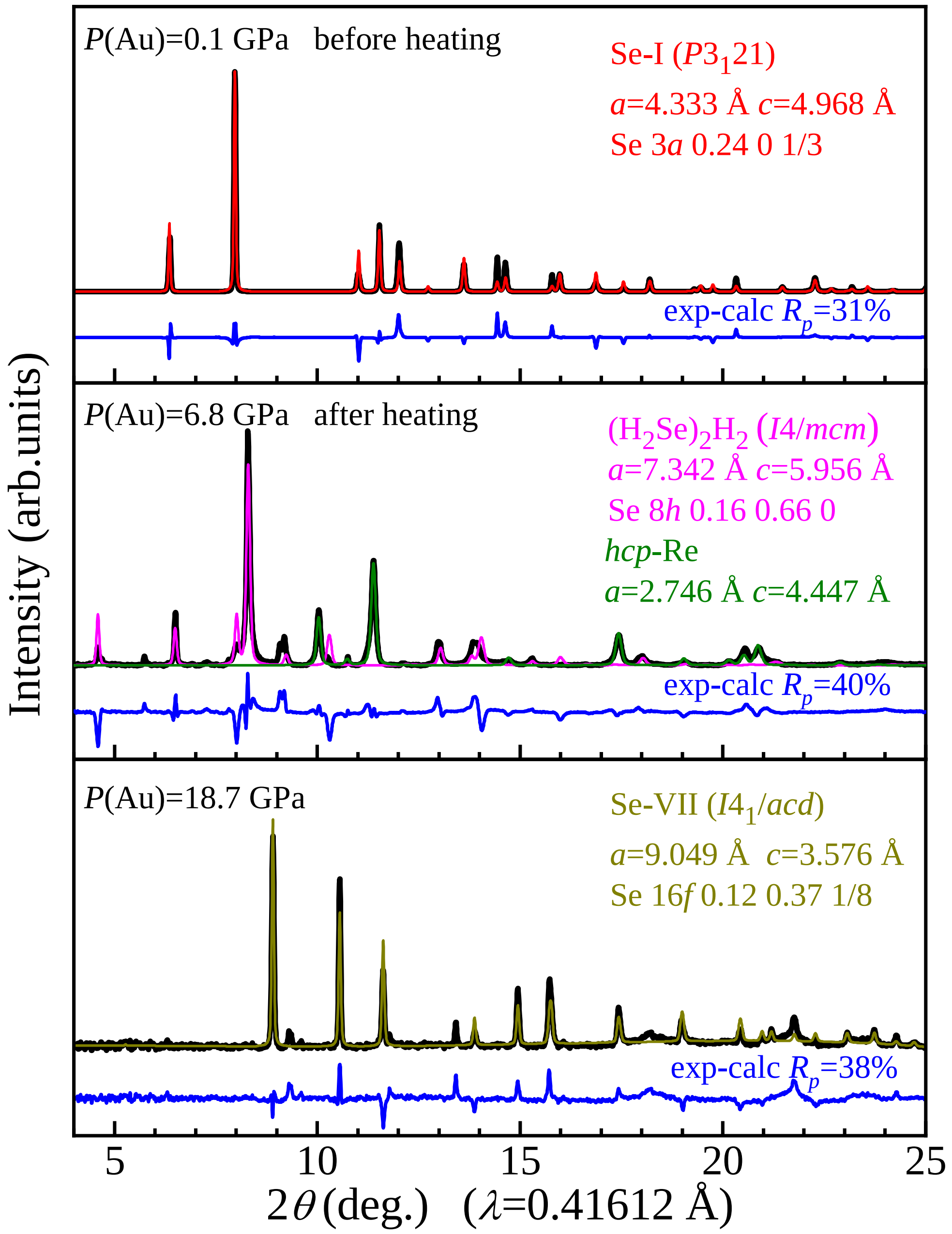

Supplementary Figure 1. Top: Representative x-ray powder diffraction pattern and the results of the Rietveld refinement of Se-I in a H_2 medium before heating at 0.1 GPa. Middle: x-ray powder diffraction pattern and the results of the Rietveld refinement upon the synthesis of $(H_2Se)_2H_2$ at 6.8 GPa. Bottom: x-ray powder diffraction pattern and the results of the Rietveld refinement after decomposition of $(H_2Se)_2H_2$ into Se-VII and H_2.

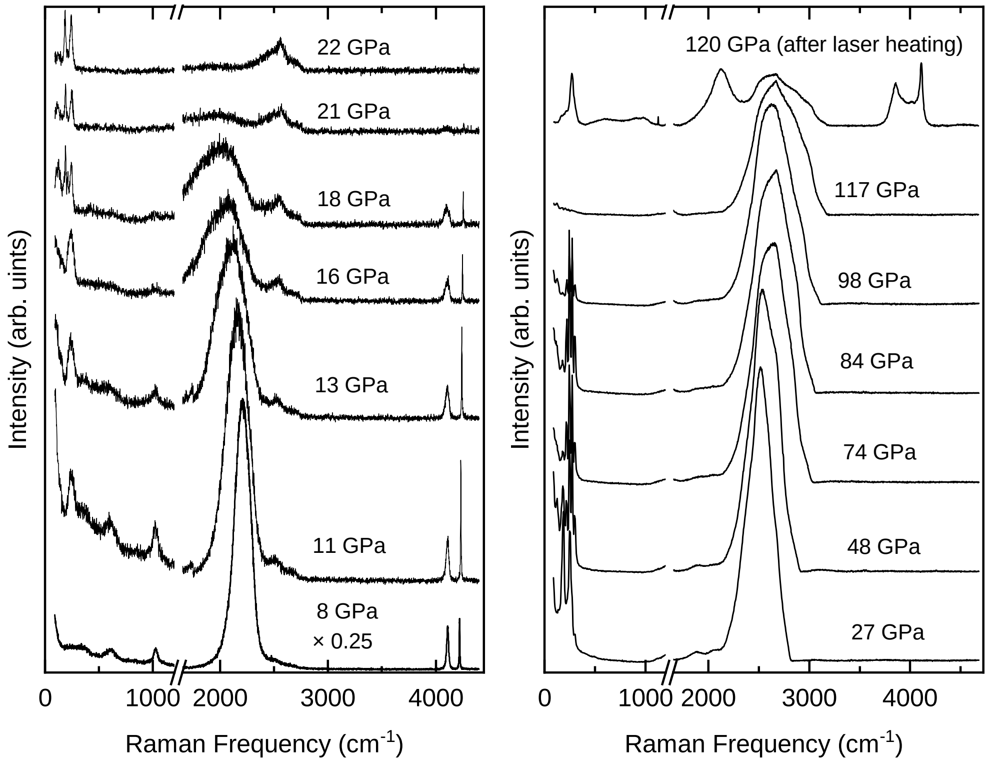

Supplementary Figure 2. Left: Representative Raman spectra as a function of pressure of $(H_2Se)_2H_2$ until decomposition into Se + H_2 above 22 GPa. Laser power was kept to below 10 mW to prevent laser induced decomposition. Right: Representative Raman spectra as a function of pressure of the decomposed sample upon compression to 117 GPa, after which the sample was laser heated to form $SeH_2(H_2)_2$.

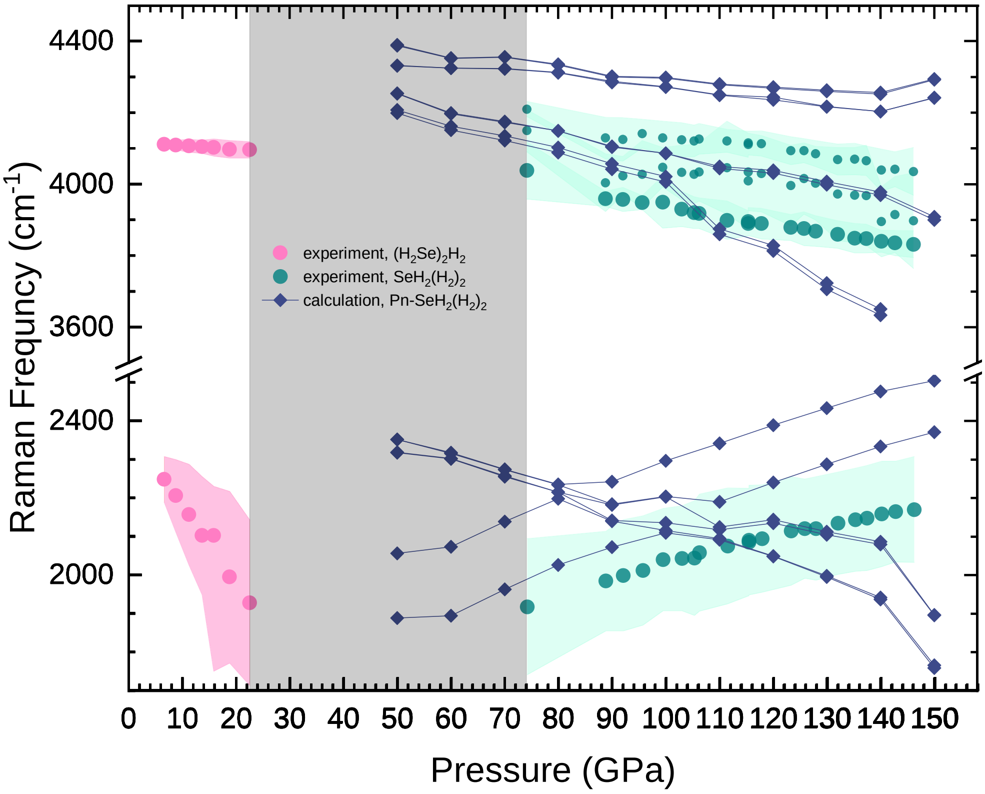

Supplementary Figure 3. Raman shift as a function of pressure of the experimentally observed Se-H modes and H-H modes compared with the calculated modes for Pn-$SeH_2(H_2)_2$ (dark blue lines and symbols). The colour shaded area represents the FWHM of the experimentally observed modes. The grey shaded area represents the pressure regime for which no Se-H compounds are observed.

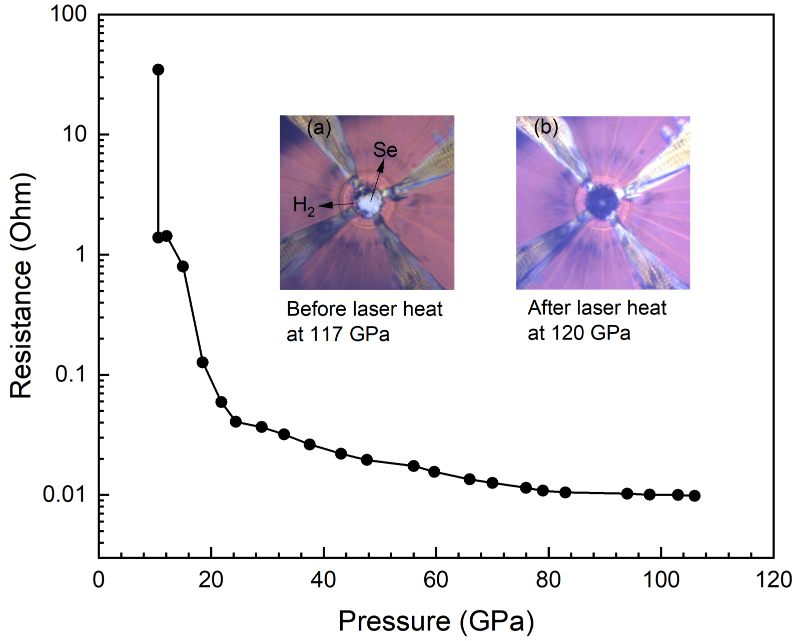

Supplementary Figure 4. Four probe electrical resistance measurements as a function of pressure of selenium in a H_2 medium. Inset (a) and (b) show micrographs of Se+H_2 before laser heat and after laser heat, respectively.

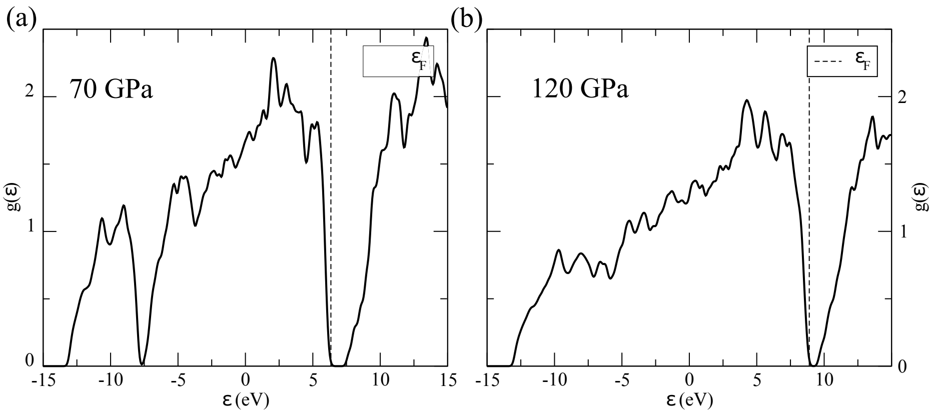

Supplementary Figure 5. Electronic density of states of Pn-H_6Se at (a) 70 GPa and (b) 120 GPa. The dashed vertical line represents the Fermi level.

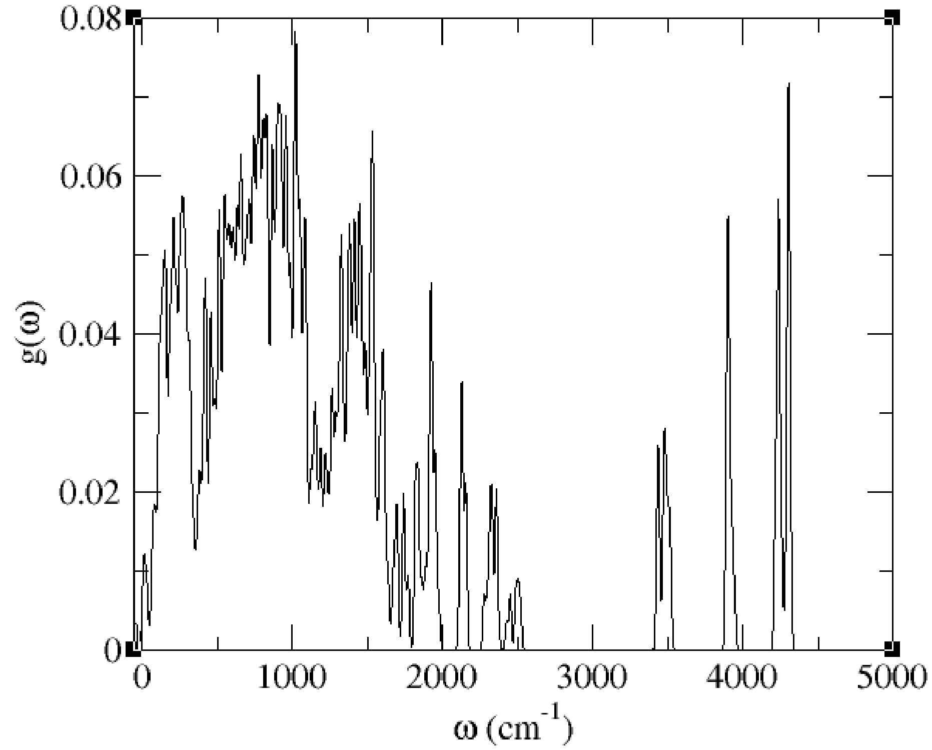

Supplementary Figure 6. Calculated phonon density of states for Pn H_6Se based on a 28 atom cell at 150 GPa, after 20 ps of molecular dynamics plus quench. Calculated frequencies are broadened by 20cm^{-1}. No imaginary modes were observed. In the quenched configuration the hydrogen molecules adopt well-defined orientation, leading to the small distortion to Pn. During the molecular dynamics at 300 K the hydrogens are rotating, which restores the $I4_1/amd$ symmetry.

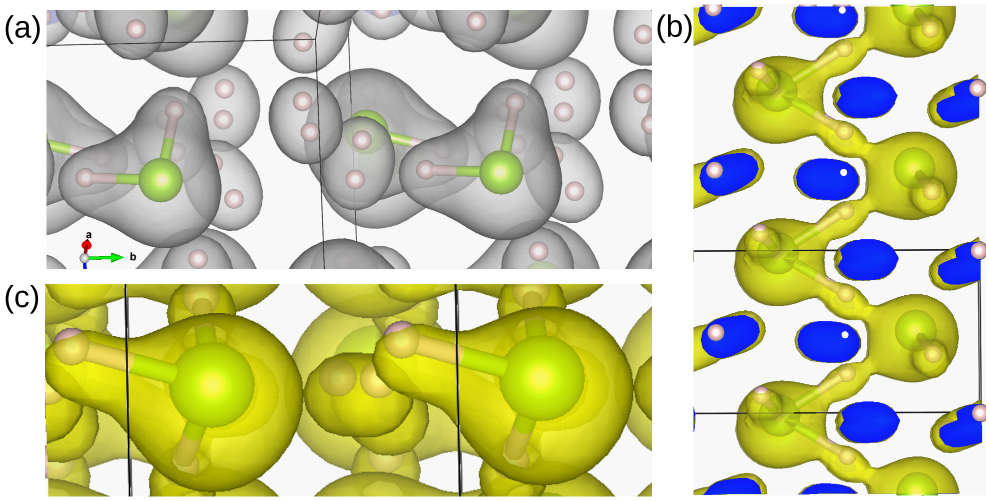

Supplementary Figure 7. (a) Charge density isosurface from DFT calculation at ambient conditions. H_6Se is not the stable phase here, but the H_2 and H_2Se molecules are distinct with 1.46-1.48Å, bondlength (b) Charge density isosurface of H_6Se at 100GPa illustrating short (1.46 Å SeH bond, with H_2 molecules also shown. (c) As b, viewed to show the chainlike bonding with long (1.58-1.60 Å), SeH bonds.

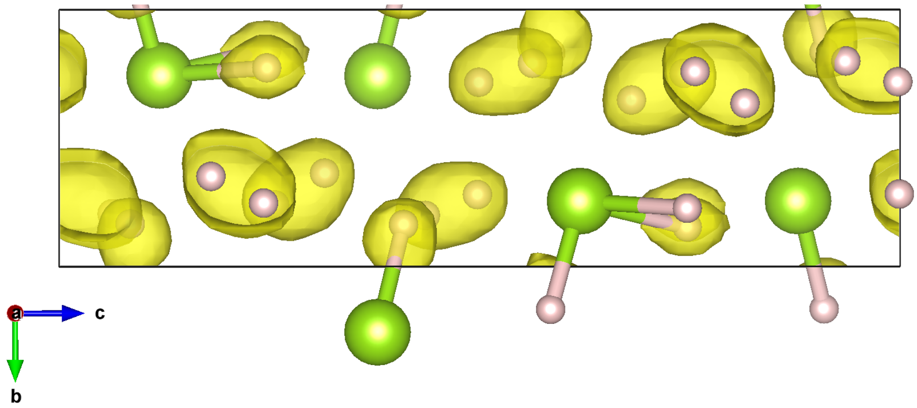

Supplementary Figure 8. Isosurface at 0.8 for the electron localisation function for H_6Se at 100 GPa calculated with CASTEP. The H_2 molecules have ELF values up to 0.98 and are clearly defined. At this ELF value there is no evidence for delocalised SeH-H-SeH chains, rather the electrons are localised on the hydrogen atoms. The Mulliken bond populations for SeH , as calculated in CASTEP, are positive (bonding) at ambient, but negative (non-bonding) at 100 GPa.

Supplementary Table 1. Birch-Murnaghan equation of state[1] parameters for $(H_2Se)_2H_2$ and $SeH_2(H_2)_2$.

Phase	V_0 (Å^3/Se)	B_0 (GPa)	B'_0
$(H_2Se)_2H_2$	54.6 (10)	13.5 (20)	4 (fixed)
$SeH_2(H_2)_2$	69 (3)	15 (2)	4 (fixed)

Supplementary Table 2. The predicted crystal structure of $SeH_2(H_2)_2$ at 100 GPa.

Pn-H_6Se 100 GPa				
Pn(No. 7) a=3.204 Å, b=3.195 Å, c=10.470 Å				
Atom	Label	x	y	z
Se	Se1	0.00310	0.25574	0.37863
Se	Se2	-0.01664	0.74563	0.61948
H	H1	0.06892	-0.20789	0.06298
H	H2	0.49538	0.28166	0.49908
H	H3	-0.02247	0.75282	0.24310
H	H4	0.49270	0.35082	0.68058
H	H5	0.40657	0.79974	0.43550
H	H6	0.99243	0.36503	0.81560
H	H7	0.48667	0.75891	0.25632
H	H8	0.24501	0.77397	0.74685
H	H9	0.60875	0.60875	0.08447
H	H10	-0.14014	0.83301	0.40954
H	H11	0.23534	0.15410	0.24683
H	H12	0.99189	0.28124	0.99740

Supplementary Table 3. Shortest Hydrogen-hydrogen and Hydrogen-selenium distances, and Mulliken bond charges between pairs of atoms at 120 GPa.For the bond charge, we use the overlap matrix of the LCAO basis between adjacent atoms [2]. Again, precise values are sensitive to details of the LCAO, but the value for non-bonded pairs are typically only 10% that of the H_2 molecules: such a strong signal is unequivocal evidence of covalent bonding. There is a consistent, if weak, trend for the transfer of charge from Se to H. Mulliken bond charges are somewhat sensitive to the choice of basis set, but it is clear that only the H_2 molecules have significant bond charges. Similar results are obtained for other pressures. Note that the shortest Se-H distances are to the non-molecular hydrogen atoms

Atom	Charge	Bondlength (Å)
H 7 – H 16	1.57	0.736
H 8 – H 15	1.57	0.736
H 3 – H 23	1.54	0.737
H 4 – H 19	1.54	0.737
H 6 – H 21	1.57	0.746
H 5 – H 22	1.57	0.746
H 1 – H 10	1.45	0.762
H 2 – H 9	1.45	0.762
H 5 – H 15	0.04	1.260
H 6 – H 7	0.04	1.260
H 10 – H 21	-0.12	1.303
H 2 – H 22	-0.12	1.304
H 9 – H 14	-0.13	1.428
H 1 – H 13	-0.13	1.428
H 11 – H 24	-0.25	1.443
H 12 – H 20	-0.25	1.443
H 6 – H 18	-0.14	1.452
H 5 – H 17	-0.14	1.452
H 10 – H 13	-0.05	1.461
H 2 – H 14	-0.05	1.461
H 1 – H 23	-0.05	1.465
H 4 – H 9	-0.05	1.465
H 17 – Se 1	0.08	1.455
H 18 – Se 3	0.08	1.455
H 14 – Se 2	-0.29	1.463
H 13 – Se 4	-0.29	1.463
H 20 – Se 1	-0.00	1.527
H 24 – Se 3	-0.00	1.527
H 11 – Se 3	0.02	1.531
H 12 – Se 1	0.02	1.531
H 24 – Se 2	0.05	1.633
H 20 – Se 4	0.05	1.633
H 11 – Se 2	0.00	1.641

Supplementary Table 4. Mulliken charges projected onto s,p,d,f orbitals at 120 GPa. Mulliken analysis is one of a number of techniques which associates electrons with localised orbitals. This starts with the Kohn-Sham one-particle orbitals for the noninteracting electrons in the plane wave basis. We then project these orbitals onto the Linear Combination of Atomic Orbitals (LCAO) basis. This gives a well-defined charge on each atom, but like all such partitionings it is highly sensitive to details of the atomic basis[3]. Notice that the bonding has no f character, and the only p,d character is on the Se atoms. Mulliken charges are somewhat sensitive to the choice of basis set[2], but it is clear that the atoms are close to neutral (one electron per hydrogen, 16 per selenium) with small charge transfer from Se to H. Similar results are obtained for other pressures.

Atom	s	p	d	Total
H 1	1.107	0.000	0.000	1.107
H 2	1.136	0.000	0.000	1.136
H 3	1.132	0.000	0.000	1.132
H 4	1.134	0.000	0.000	1.134
H 5	1.129	0.000	0.000	1.129
H 6	1.129	0.000	0.000	1.129
H 7	1.136	0.000	0.000	1.136
H 8	1.132	0.000	0.000	1.132
H 9	1.107	0.000	0.000	1.107
H 10	1.135	0.000	0.000	1.135
H 11	1.118	0.000	0.000	1.118
H 12	1.118	0.000	0.000	1.118
H 13	1.080	0.000	0.000	1.080
H 14	1.080	0.000	0.000	1.080
H 15	1.136	0.000	0.000	1.136
H 16	1.132	0.000	0.000	1.132
H 17	1.101	0.000	0.000	1.101
H 18	1.101	0.000	0.000	1.101
H 19	1.132	0.000	0.000	1.132
H 20	1.106	0.000	0.000	1.106
H 21	1.118	0.000	0.000	1.118
H 22	1.119	0.000	0.000	1.119
H 23	1.134	0.000	0.000	1.134
H 24	1.106	0.000	0.000	1.106
Se 1	1.203	3.971	10.003	15.176
Se 2	1.314	4.077	10.002	15.394
Se 3	1.203	3.971	10.003	15.176

Supplementary References

[1] Birch, F. Finite elastic strain of cubic crystals. *Phys. Rev.* **71**, 809–824 (1947). URL https://link.aps.org/doi/10.1103/PhysRev.71.809.

[2] Segall, M. D., Shah, R., Pickard, C. J. & Payne, M. C. Population analysis of plane-wave electronic structure calculations of bulk materials. *Phys. Rev. B* **54**, 16317–16320 (1996). URL https://link.aps.org/doi/10.1103/PhysRevB.54.16317.

[3] Davidson, E. R. & Chakravorty, S. A test of the hirshfeld definition of atomic charges and moments. *Theoretica chimica acta* **83**, 319–330 (1992). URL https://doi.org/10.1007/BF01113058.
